# Gliomatosis cerebri presenting as rapidly progressive dementia and parkinsonism in an elderly woman: a case report

**DOI:** 10.1186/1752-1947-2-53

**Published:** 2008-02-20

**Authors:** Emmanuelle Duron, Anne Lazareth, Jean-Yves Gaubert, Carole Raso, Olivier Hanon, Anne-Sophie Rigaud

**Affiliations:** 1 Department of Geriatrics, University René Descartes. Broca Hospital, AP-HP, France

## Abstract

**Introduction:**

Dementia is one of the most important neurological disorders in the elderly. Dementia of tumoral origin is rare and parkinsonism of neoplastic origin is unusual. We herein report a case of gliomatosis cerebri, a very rare brain tumor seldom affecting the elderly, which presented as rapidly progressive dementia and parkinsonism.

**Case presentation:**

An 82-year-old woman very rapidly developed progressive dementia and akineto-rigid parkinsonism. Brain CT scan was normal. Cerebral magnetic resonance imaging (MRI) with gadolinium injection highlighted a diffuse tumor-related infiltration involving both lobes, the putamen, the pallidum, the substantia nigra, and the brainstem, corresponding to the specific description and definition of gliomatosis cerebri.

**Conclusion:**

This atypical presentation of a gliomatosis cerebri, and the infiltration of the substantia nigra by the tumor, merits attention.

## Introduction

Dementia is one of the most important neurological disorders in the elderly.

In occidental countries, the most common forms of dementia are Alzheimer's disease and vascular dementia, with frequencies of 70 and 15%, respectively. Dementia of tumoral origin is rare. It may be related either to the tumour itself, especially primary central nervous system lymphoma or low grade glioma, or to the tumour's treatment (radiation-induced encephalopathy). Secondary parkinsonism is frequent among elderly people. It includes drug-induced parkinsonism (due to Dopamine Receptor Blockers) and vascular parkinsonism. Nevertheless, parkinsonism of neoplastic origin is unusual. We herein report a case of probable gliomatosis cerebri, a very rare brain tumor seldom affecting the elderly, which presented as rapidly progressive dementia and parkinsonism.

## Case presentation

**Figure 1:**
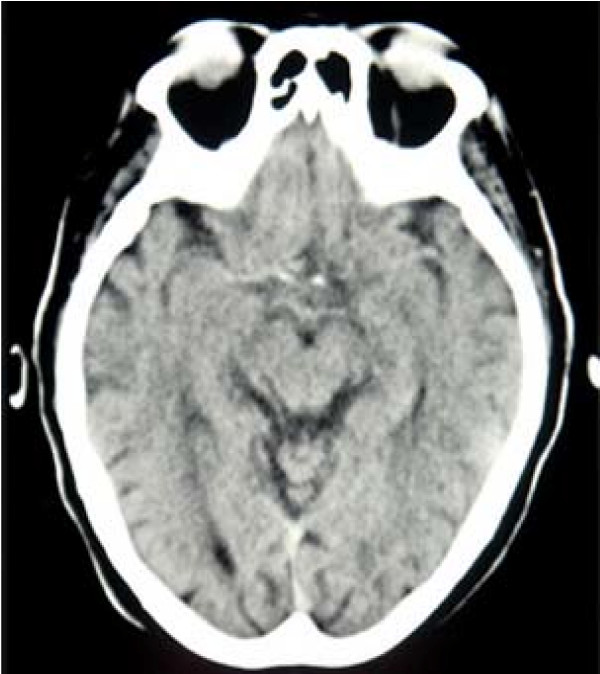
Normal non-injected computed tomography brain scan.

Following a fall, an 82-year-old woman was admitted to the Broca University Hospital. According to her family, she had exhibited cognitive impairment for several months. The patient was undergoing treatment for hypertension with Candesartan (Angiotensin Receptor Blockers) and her type 2 diabetes was satisfactorily controlled by diet. The physical examination at the time of admission revealed an akineto-rigid bilateral, symmetrical parkinsonism. Her gait demonstrated marked reduction in arm swing. She displayed bilateral bradykinesia, limb rigidity and hypomimia. Her voice was monotonous and hypophonic. There was neither tremor nor orthostatic hypotension. She scored 18/30 on the Mini-Mental Status Examination (MMSE) [1] and scored very low on the Cognitive Efficiency Profile [2], a complete validated comprehensive cognitive battery assessment, indicating a major dysexecutive syndrome (perseveration, judgment trouble, confabulation, anosognosia and apathy) and memory impairment (short and medium recall). There were also deficits on tests of visuospatial ability. Conversely, naming was preserved. Basic biological screening tests (i.e. blood cell count, blood chemistry, C-reactive protein, thyroid stimulating hormone, vitamin B12 and folic acid) were normal, as well as a non-injected computed tomography brain scan (Figure 1). An electro-encephalogram demonstrated slow waves, especially at the level of the left temporal lobe. Allowing for this dementia with parkinsonism, the first diagnostic hypothesis was dementia with Lewy bodies.

Shortly following her evaluation, the akinesia and rigidity worsened, and a frontal syndrome developed. An L-dopa treatment was introduced as well as an anticholinesterase treatment (Galantamine) without any improvement noted.

**Figure 2:**
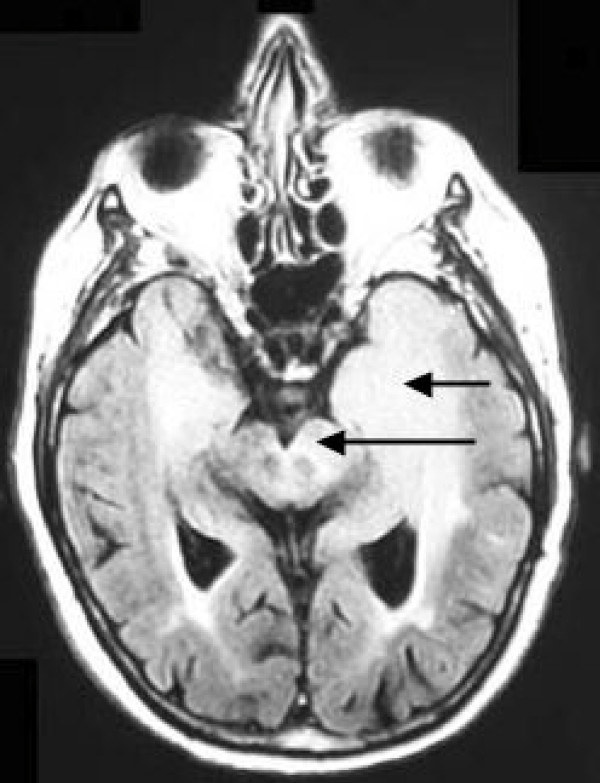
Axial fluid-attenuated inversion recovery MRI image demonstrating tumor-related infiltration involving both temporal lobes (Short arrow), and the substantia nigra (Long arrow).

**Figure 3:**
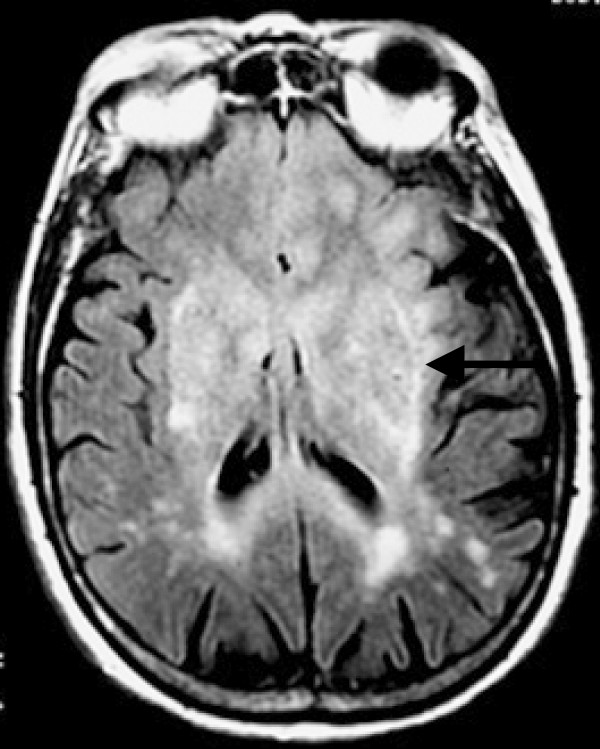
Axial fluid-attenuated inversion recovery MRI image demonstrating tumor-related infiltration involving lenticular nuclei (Arrow).

Two months after the hospital admission, the MMSE score was 5/30. Cerebral magnetic resonance imaging (MRI) with gadolinium injection highlighted a diffuse tumor-related infiltration involving both lobes, the putamen, the pallidum, the substantia nigra and the brainstem, corresponding to the specific description and definition of gliomatosis cerebri [3] (Figures 2 and 3).

Fifteen days later, the patient died of urinary sepsis just before the initiation of chemotherapy.

## Discussion

The association of parkinsonism, falls and dementia is suggestive of a dementia with Lewy bodies [4]. Indeed, very rapidly progressing dementias with Lewy bodies have been described [5,6]. However, the lack of recurrent hallucinations, delusions and fluctuating cognition and the absence of treatment response to treatment did not favour this diagnosis in our patient [4]. Furthermore, a diagnosis of multiple system atrophy was also considered, but rejected because of the very rapid course of the disease [7]. The MRI with gadolinium injection highlighted typical images of gliomatosis cerebri (i.e., diffusely infiltrative gliomas, without an obvious tumor mass, involving more than two lobes and extending to an infratentorial structure [3]), whose topography was consistent with this patient's symptoms.

Cognitive disorders are rarely caused by brain tumours but they have been reported as relatively common symptoms of primary brain lymphomas, low grade gliomas, or gliomatosis cerebri [8].

Parkinsonism of neoplastic origin is also rare. Usually this is due to tumors not involving the basal ganglia, such as astocytomas, meningiomas, craniopharyngiomas, colloid cysts, and less frequently, metastases [9].

On the other hand, tumours of the basal ganglia are rarely accompanied by parkinsonism [9], which is why brain lymphomas are frequently seen to involve basal ganglia, but without symptoms of parkinsonism [10].

Moreover, in our patient, the MRI with gadolinium injection highlighted lesions of gliomatosis cerebri with the rarely observed involvement of the substantia nigra (Figure 2). This topography explained the symptoms of, parkinsonism at least in part since the pallidum was also involved (Figure 3), with presynaptic dysfunction of the nigro-striatal pathway. Moreover, lesions of the connecting fibers in the white matter, implicated in frontal-subcortical circuits, must have contributed to the development of parkinsonism and dementia in this patient. Some small nodes were enhanced with gadolinium injection.

Gliomatosis cerebri is a rare tumor. An extensive review encompassed 22 cases with a mean occurrence age of 49 years [3]. The main symptoms are dementia, seizures, and hemiparesis. To our knowledge, the symptoms affecting our patient have been reported only in three other cases [11-13], but this case is noteworthy in having an atypical symptomatology and also the first reported lesion in the substantia nigra, as shown by MRI. Nevertheless, although the MRI images are typical of gliomatosis cerebri, a limitation of this case report is the lack of pathological confirmation of the diagnosis.

## Conclusion

This atypical presentation of a gliomatosis cerebri, and the infiltration of the substantia nigra by the tumor, merits attention.

## Competing interests

The author(s) declare that they have no competing interests.

## Authors' contributions

All authors participated in the care of the patient described. ED wrote the manuscript. AL, JYG, CR collected data and helped to draft the manuscript. OH and ASR critically revised the content of the manuscript. All authors have read and approved the final version of the manuscript.

## Consent

Written informed consent was obtained from the next of kin of the patient described in this case report for publication of this case report and the accompanying images. A copy of the written consent is available for review by the Editor-in Chief of this journal.
